# Selenium supplementation influences mice testicular selenoproteins driven by gut microbiota

**DOI:** 10.1038/s41598-022-08121-3

**Published:** 2022-03-10

**Authors:** Sara Ramírez-Acosta, Marta Selma-Royo, María Carmen Collado, Francisco Navarro-Roldán, Nieves Abril, Tamara García-Barrera

**Affiliations:** 1 Department of Chemistry, Faculty of Experimental Sciences, Research Center of Natural Resources, Health and the Environment (RENSMA), Campus El Carmen, University of Huelva, Fuerzas Armadas Ave., 21007 Huelva, Spain; 2 Department of Biotechnology, Institute of Agrochemistry and Food Technology-National Research Council (IATA-CSIC), Agustin Escardino 7, 46980 Paterna, Valencia Spain; 3 Department of Integrated Sciences, Cell Biology, Faculty of Experimental Sciences, University of Huelva, Huelva, Spain; 4 Department of Biochemistry and Molecular Biology, University of Córdoba, Campus de Rabanales, Edificio Severo Ochoa, 14071 Córdoba, Spain

**Keywords:** Chemical biology, Metals, Proteins, Microbiome

## Abstract

Selenium is a well-known essential element with important roles in human reproductive health mainly due to its antioxidant character. This study aimed to investigate the potential role of selenoproteins on gut microbiota and male reproductive health. A new assay for the absolute quantification of selenoproteins in testicular tissue based on two dimensional chromatography with inductively coupled plasma mass spectrometry was performed for the first time. The gut microbiota profile was obtained by 16S rRNA gene sequencing. Numerous associations were found between testicular selenoproteins and gut microbiota (e.g.* Mucispirillum*, related with sperm activity and testosterone, was associated with glutathione peroxidase (GPx) and selenoalbumin (SeAlb), while *Escherichia/Shigella*, related to sex hormones, correlated with GPx, selenoprotein P (SelP) and SeAlb). The effects of Se-supplementation on testicular selenoproteins only occur in conventional mice, suggesting a potential selenoproteins-microbiota interplay that underlies testicular function. The selenoproteins GPx and SelP have been quantified for the first time in the testicles, and the novel identification of SeAlb, a protein with nonspecifically incorporated Se, is also reported. These findings demonstrate the significant impact of Se-supplementation on gut microbiota and male reproductive health. In addition, the analytical methodology applied here in selenoprotein quantification in testicular tissue opens new possibilities to evaluate their role in gut microbiota and reproductive health axis.

## Introduction

Selenium (Se) is an essential trace element with important roles in immune function, the metabolism of thyroid hormones^1^ and cancer chemoprevention^2^. Se deficiency has been related to heart failure, nutritional myodegeneration (white muscle disease)^3^ and Keshan disease^4^ among other pathologies. Se dietary supplementation leads to the formation of specific selenoproteins, in which Se occupies the active center; hence, influencing the redox-regulated genes and helping the cell to convert with reactive oxygen species (ROS) into less reactive molecules^5^. Se is also important in reproductive health^6^, being essential for gonadal development, gametogenesis and fertilization^7^, likely as a result of its ability to modulate antioxidant defense mechanisms and redox sensitive pathways. There is clear evidence that a deficiency of Se and selenoproteins can lead to several reproductive health and obstetric complications as well as infertility, preeclampsia, miscarriage, preterm labor, fetal growth restriction, gestational diabetes and obstetric cholestasis^6^. Mammalian Se-containing proteins can be divided into three groups: (1) proteins containing non-specifically incorporated Se, in which sulfur is replaced by Se in amino acids such as methionine (SeMet) (e.g. selenoalbumin (SeAlb), which is not considered as a “real” selenoprotein), (2) specific Se-binding proteins (e.g. Se-binding protein 1, SBP1), in which Se is tightly associated with a cysteine (Cys) residue in the peptide but not as a component of selenocysteine (SeCys) and (iii) specific selenocysteine-containing selenoproteins (e.g. selenoprotein P (SELENOP))^8^. The role of Se in mammal spermatogenesis is mainly mediated by two selenoproteins, namely phospholipid hydroperoxide glutathione peroxidase (PHGPx/GPx4) related to sperm quality and male fertility and SELENOP, a plasma protein required for Se supply to the gonads where it is used as a reservoir of Se^9^. Other selenoprotein transcripts (~ tenfold lower level than PHGPx and the majority of them with unknown function) have also been identified in male gonads (Thioredoxin/Glutathione Reductase (TGR), selenoprotein V, selenoprotein W, selenoprotein K, selenoproteins 15 and selenoprotein S)^9^.

Recent studies have pointed out the potential role of dietary Se in shaping the gut microbiota and, subsequently, exerting effects on host metabolism and immunity^10–13^. Diet is considered a key regulator of gut microbiota with effects at local and systemic levels^14,15^ and also, on reproductive hormones levels^16^. Several reports have shown that high-fat diet induced gut dysbiosis can affect the system’s health even causing neurological disorders^17,18^ and spermatogenesis impairments^19^. Recent studies suggest the impact of gut microbiota on fertility and reproductive health in both, males and females^20–22^. However, little is known about the mechanisms underlying the shifts in gut microbiota across reproductive states. An increase in dietary Se intake has also been implicated in enhancing the antioxidant GPX activity, thereby improving male fertility^23^. The encouraging results in the last years suggest that the combination of Se with other essential micronutrients may improve reproductive efficiency in males^23^. However, to date there is not sufficient nor consistent findings upon which to draw solid conclusions. Indeed, previous studies regarding selenoproteins in the testicles applied non quantitative methods, such as transcriptomics or enzymatic assays^9^.

The aim of this work is to investigate the potential role of selenoproteins in the gut microbiota-reproductive health axis. For this purpose, the absolute quantification of selenoproteins by intact protein analysis will be performed using a metallomic approach based on inductively coupled plasma mass spectrometry (ICP-MS), a methodology used for analyses of serum, plasma^24^ and the liver^25^. To this end, mice testicular selenoproteome has been determined after Se-supplementation of conventional mice and mice with microbiota depleted by antibiotics. The total metal content in testicles has been also measured to evaluate the possible impact of Se-supplementation and microbiota on the homeostasis of elements in testicles, as well as their traffic.

## Results and discussion

### Preliminary observations and histopathology evaluation

This study analyzed the impact and effect of a Se-enriched diet on selenoproteins, total Se concentration and metal homeostasis in the testes for 8 week-old male *Mus musculus* mice as well as the relationships of these parameters with the gut microbiota composition. To study the influence on the gut microbiota, half of the mice received a cocktail of antibiotics (200 mg kg^−1^ per body weight (bw) of ampicillin, neomycin and metronidazole, 100 mg kg^−1^ bw of vancomycin and 2 mg kg^−1^ bw of amphotericin B)^26^ for one week, they were later fed with either a regular or a Se-supplemented diet for a further weeks. The Se-enriched diet provided the mice with a daily intake of 120 µg kg^−1^ bw, three times the regular mouse intake of Se^27^. This dose of Se is no-toxic, but able to modify some biological parameters^28^. The animals showed no external evidence of illness or discomfort, and all survived the treatment. No differences were found between the body weights of the mice at the end of the treatment. However, pretreatment with Abx caused a decrease in the testicular weight of mice (Fig. S2), albeit statistically non-significant one.

**Figure 1:**
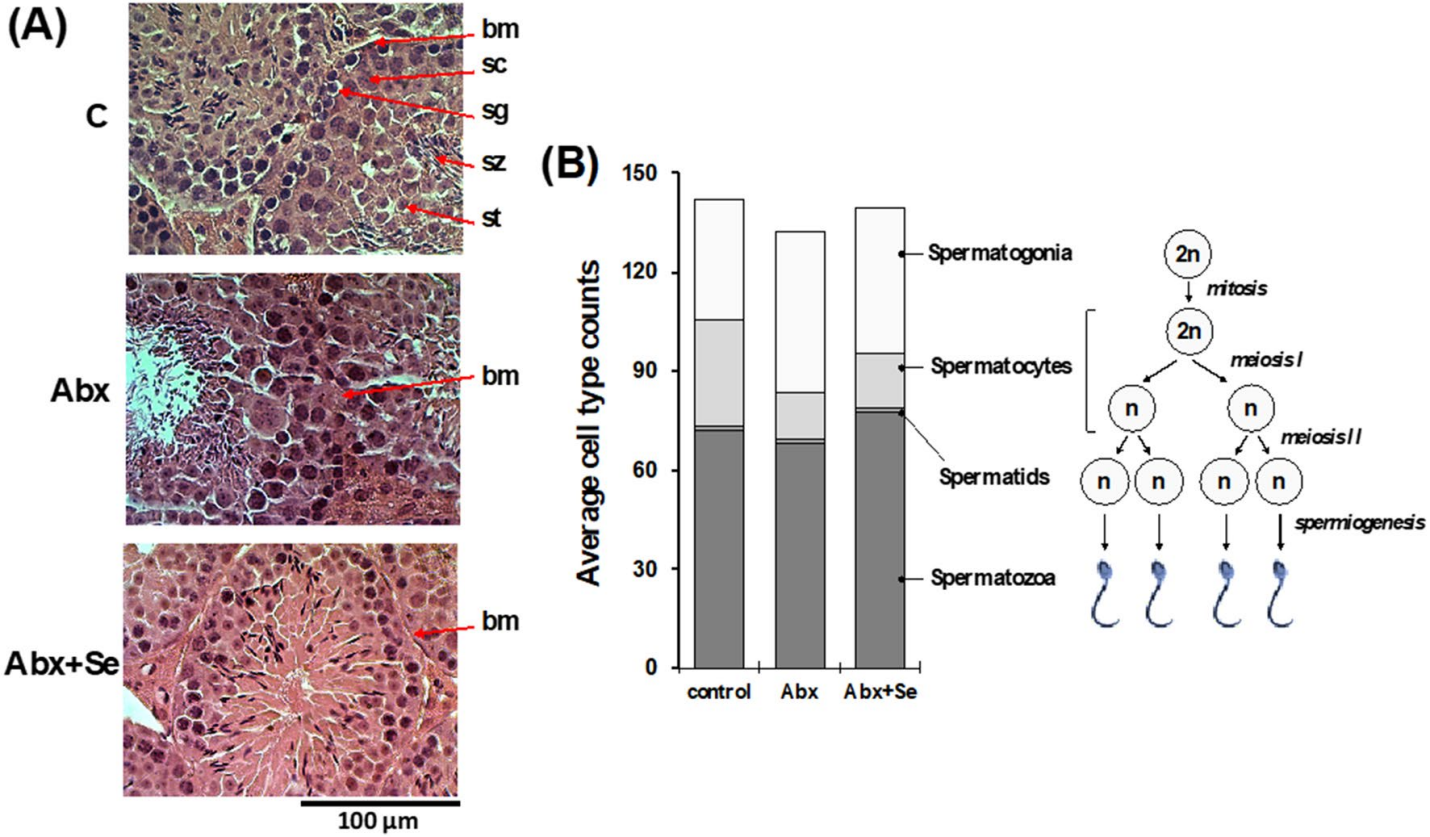
(**A**) Photomicrograph showing the cross section of H&E staining of the testes of a representative mouse from groups Control, C-Se, Abx and Abx + Se. The basement membrane (bm), spermatogonia cells (sg), spermatocytes (sc), spermatids (st) and spermatozoids (sz) are indicated by arrows. (400 × magnification). (**B**) Bar plots showing the average counts for each germ cell type in the seminiferous tubule; the scheme illustrates the process of generation of the different types of germ cells during spermatogenesis.

Studies in animal models clearly demonstrate the deleterious effects of antibiotics on testicular function^29^. The antibiotic cocktail we used here to deplete the intestinal microbiota included the aminoglycoside neomycin, an antibiotic that adversely affects spermatogenesis by cessation of meiosis at the level of primary spermatocytes^30^. Our histopathological study confirms this effect (Fig. 1), as we found a greater number of spermatogonia, the diploid undifferentiated germ cells, in the seminiferous tubules of the Abx group. Although the differences between the Abx group and the control group were not statistically significant, these results suggest a possible arrest of the cell division processes that convert spermatogonia into spermatozoa. The Abx group’s histological samples also showed an alteration of the normal structure of the seminiferous tubes with mild and punctual degenerative changes at the level of the basement membrane. This morphological layout is essential for the process of spermatogenesis since it is the structural and hormonal support of the spermatogonia in the different stages of the seminiferous epithelial cycle^31^ and abnormal basement membrane structures have been associated with spermatogenesis^32^. Although no significant decrease in the number of sperm per seminiferous tube was observed in any sample, our data suggests that Abx treatment may alter male fertility. The intake of a Se supplement after pretreatment with Abx (Abx-Se group) did not completely prevent the effect of Abx on the meiotic process but prevented the basement membrane abnormalities observed in the Abx group. These results suggest that Se supplementation likely improves the fertility of mice, in agreement with previous reports^9^.

### Selenoproteins and total selenium in testicles

**Figure 2:**
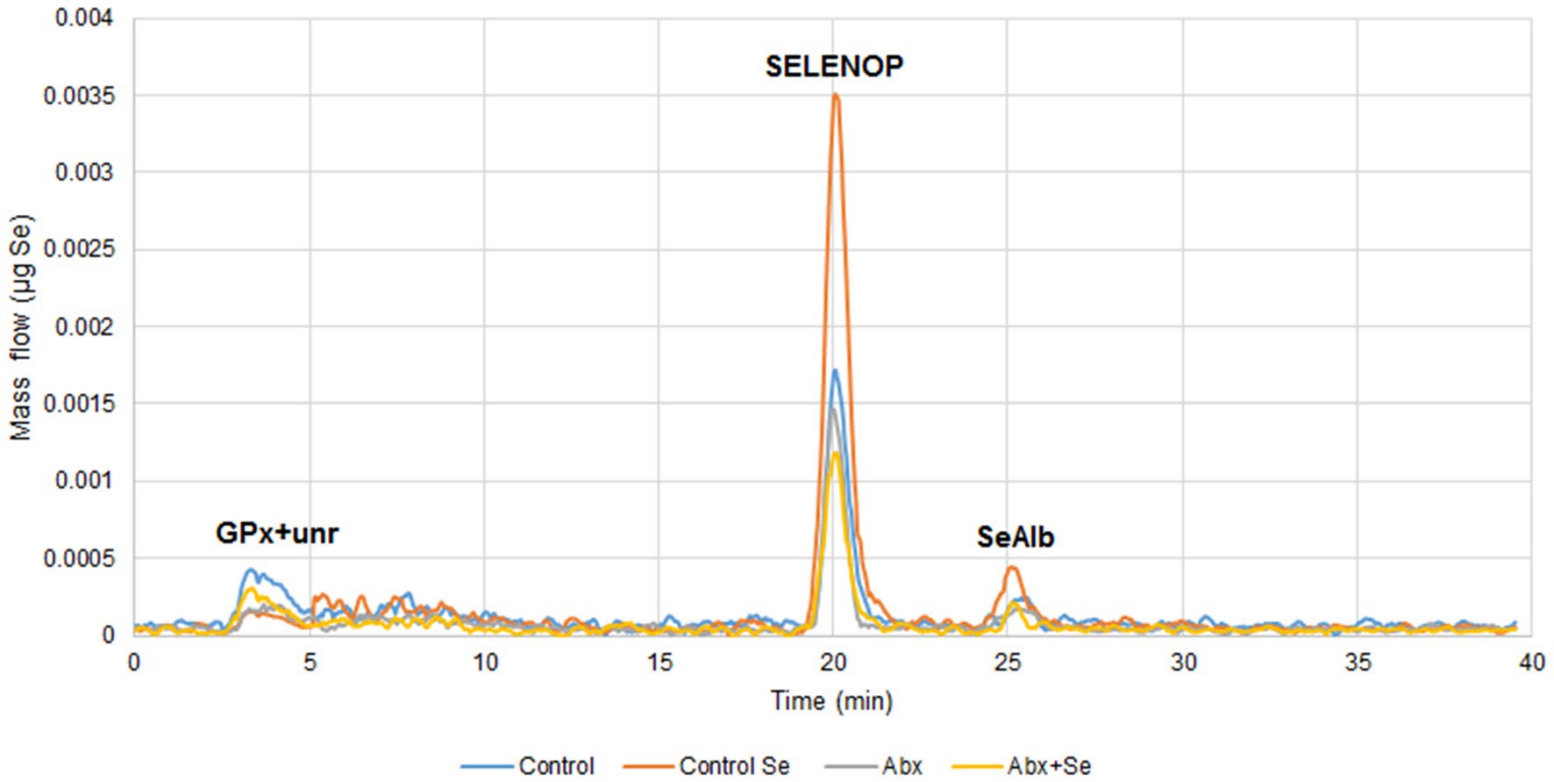
Selenium mass flow chromatogram obtained from testicular tissues of mice.

Selenoprotein extraction protocols from mammalian tissues requires high efficiency, avoiding interferences and protein degradation during the procedure. For total protein extraction we used here the CelLytic™ MT extraction reagent for total protein extraction, which contains a low concentration of a dialyzable mild detergent for minimal interference with protein interactions and biological activity^33^. Selenoproteins obtained from mice testicles from each study group were clearly identified in the typical mass flow chromatogram (Fig. 2). These chromatograms represent the mass of Se (µg) vs time (min), and, thus, they do not reflect the abundance of different selenoproteins, but the Se accounted for by each one. When converting chromatograms peaks to selenoproteins abundance, it must be taken into consideration that both mice and human SELENOP contains 10 selenocysteine molecules (C_3_H_7_NO_2_Se)^34^, while GPx has 4 g atoms of Se per mole^35^. In addition, we quantified SeAlb, which is not a “real selenoprotein”, but a protein that incorporates Se post-translationally in the form of selenomethionine (SeMet)^36^. Furthermore, the relative concentration of selenoprotein (in terms of Se) in the testicles is SELENOP > GPx + unretained (unr) ~ SeAlb (Fig. 2). It is noteworthy that GPx elutes in the void of the column and this peak should be assigned to GPx and other unretained selenoproteins (they have not been identified in mice testicles by organic mass spectrometry and will be the object of future work).

**Figure 3:**
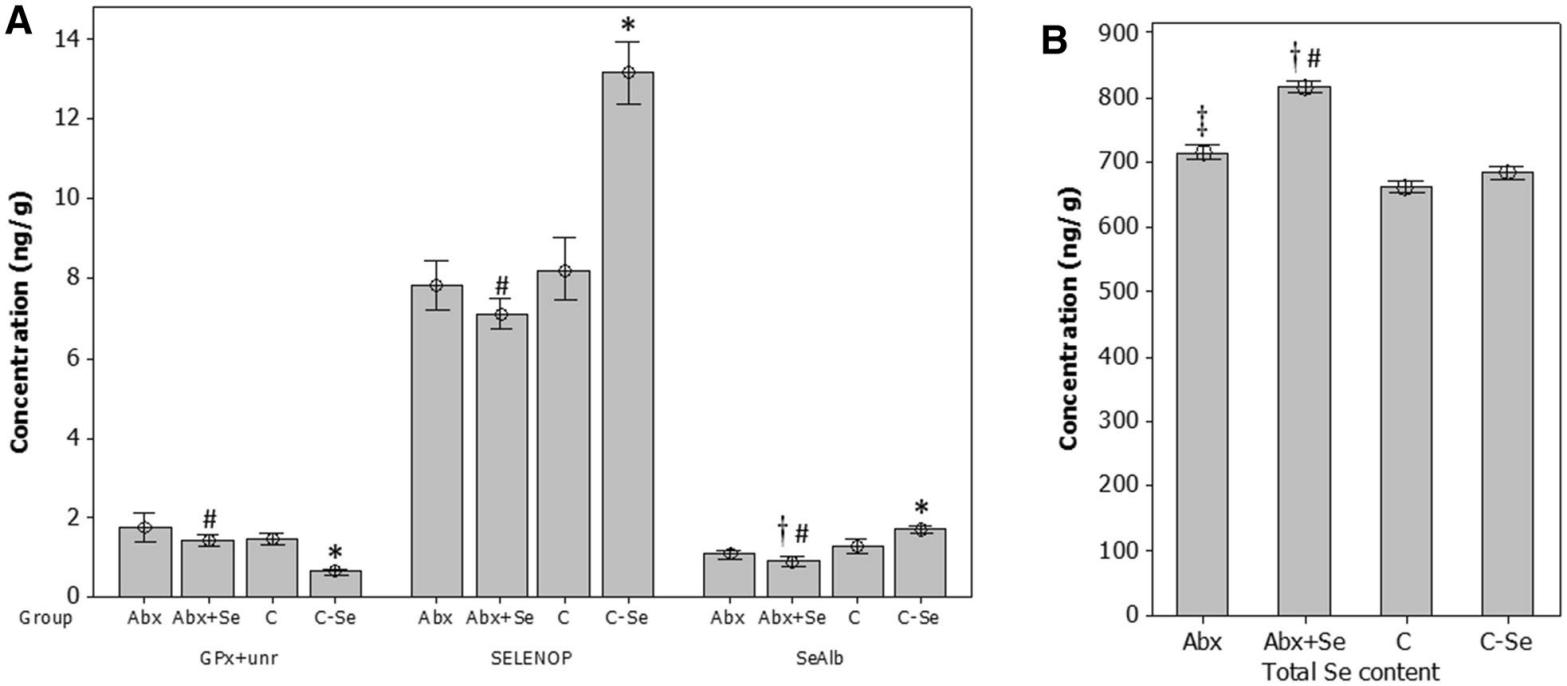
Selenoproteins (**A**) and total Se concentration (**B**) in the mouse testis. Data are expressed as mean ± SD (n = 10). (*) Statistically different in C-Se vs C comparison. (†) Statistically different in Abx + Se vs C comparison. (#) Statistically different in Abx + Se vs C-Se comparison. (‡) Statistically different in Abx + Se vs Abx comparison.

Significant changes in both selenoprotein and total Se concentrations were observed between groups (Fig. 3 and Table S1). The total Se content in the testicles is affected by Se-supplementation. In conventional mice fed with a Se-supplemented diet (C-Se group), the total Se content was similar to the control group, and there were no statistical differences between groups. In contrast, mice fed Se-supplemented diet after microbiota depletion (Abx + Se group) presented the highest concentrations of Se in the testicles, showing statistical differences with the control group (C, 1.24-fold↑, *p* = 0.01), with the Se-supplemented conventional mice (C-Se, 1.19-fold↑, *p* = 0.005) and with the microbiota-depleted mice that were fed a rodent diet (Abx, 1.15-fold↑, *p* = 0.01).

Regarding selenoproteins, the concentrations of SELENOP and SeAlb were highest in the testicles of conventional mice fed a Se-supplemented diet, while the concentration of GPx + unr was the lowest in this group. Thus, Se supplementation increases the concentration of SELENOP (1.61-fold↑, *p* = 0.000) and SeAlb (1.30-fold↑, *p* = 0.013) in the testicles of conventional mice (C-Se vs C), while decreasing the concentration of GPx + unr (2.32-fold↓, *p* = 0.000). The SeAlb concentration in the testicles was significantly different between Abx + Se and C groups (1.54-fold↓, *p* = 0.017). Se-supplementation of microbiota depleted mice (Abx + Se vs C-Se) increased the concentration of GPx + unr in testicles (2.22-fold↑, *p* = 0.000) and decreased that of SELENOP (1.85-fold↓, *p* = 0.000) and SeAlb (2.00-fold↓, *p* = 0.000). As mentioned in a previous study PHGPx/GPx4 (most abundant) and SELENOP have been previously identified in testicles along with other proteins with unknown function or lower abundance^8^. However, to our knowledge, this is the first identification of SeAlb in the testicles, likely due to either the methodologies previously used (transcriptomics and enzymatic activities^37,38^ or a lack of information about its presence and role in the testicles. Testosterone is produced by the Leydig cell and secreted into the interstitial fluid from where it is taken up by the Sertoli or diffuses into the interstitial capillaries to bind to albumin for transport through the body. If the presence of Se in albumin favors testosterone binding and distribution to other organs and tissues, this is an interesting issue to be addressed in further studies. In addition, the absolute quantification of selenoproteins in the testicles has not been reported before. Moreover, the selenoproteins determined in this work accounts for the highest Se content linked to proteins in the testicles as concluded from the mass flow chromatogram (Fig. 2). Selenometabolites (SeO_4_^2−^, SeO_3_^2−^, SeMet, SeCys, SeMetSeCys) that elute between GPx and SELENOP were under the detection limits in all analyzed samples (LD = 0.5 ng Se g^−1^).

Thus, we suggest that Se-supplementation in conventional mice influences the selenoprotome, but not the total concentration of Se in the testicles (Fig. 3). Indeed, SELENOP and SeAlb patterns are parallel, as they both increase in concentration after Se-supplementation. However, the concentration of GPx + unr decreased in the testicles in Se-supplemented conventional mice. Nevertheless, Se-supplementation of microbiota-depleted mice (Abx + Se vs*.* Abx) has no effect on the testicular selenoproteome, but the total concentration of Se is significantly increased. These findings suggest that the effect of Se-supplementation on the selenoproteome of the testicles could be influenced and mediated by microbiota although the exact mechanisms remain unknown.

Se-supplementation has proven the beneficial effects of Se in male fertility reproduction and testicular damage^23,39–41^.The main function of SELENOP is the transport and distribution of Se to other tissues, but it also possesses antioxidant action and it is involved in Se homeostasis^42^, while the GPx family are antioxidant selenoproteins^43^ and SeAlb is a Se transporter^44^. In the testicles, SELENOP is located in the Leydig cells^45^ and can influence sperm quality and, hence, male fertility^38,46^. PHGPx/GPx4 has also been related to sperm midpiece, mitochondrial sheath and sperm chromatin condensation^8^.

In previous work, the expression levels of GPx and SELENOP in the testicles of mice were not affected with dietary Se deficiency or excess selenomethionine^47^, however, in rats with Se deficiency the expression levels of SELENOP were decreased^37^. Other authors reported that Se-supplementation sharply increase the activity of testicular SELENOP^46^ and, in studies of co-exposure to Cd and Se, Se ameliorates the effects of Cd by increasing SelP and GPX4 gene expression^48^. The results obtained from studies of Se-supplementation in human prostate adenocarcinoma cells (F-9 and Du-145 cells) revealed an increase in mRNA expression levels on the glutathione peroxidases GPX1, GPX2 and GPX3, SelS and SEP15, while some selenoproteins located in the testes, such as SelW and SelV changes slightly and the TRXR3 selenoprotein decreased sharply^49^.

To summarize, our results suggest that the use of antibiotics (Abx) may affect the ability of the host to incorporate Se into SELENOP, which, as we have mentioned, may influence testicular activity and reproductive function, in good agreement with the above results.

Metals and metalloids are very important in biology since as one-third of all proteins in the human body require a metal cofactor for functionality^50^. Metallomics can be defined as the research field that elucidates the identification, distribution, dynamics, role and impact of metals and metalloids in biological systems^51,52^. The methodology for a metallomic analysis usually involve the use of an inductively coupled plasma mass spectrometer (ICP-MS) hyphenated to high performance liquid chromatography (HPLC), gas chromatography (GC–MS) or capillary electrophoresis (CE)) using the heteroelement (an atom different to C, H, N, O or F, e.g. Se) in the biomolecule as a “tag” (heteroatom-tagged proteomics)^53^. Thus, this approach, is more sensitive than the typical proteomic approaches which involve tryptic digestion and further analysis of peptides that are usually difficult to separate and it has not been previously applied for the absolute quantification of selenoproteins in testicular tissue^54^. Other techniques such as UV–Vis spectrophotometry allow determination of the total content of proteins or their activities, but not the absolute quantification of specific proteins.

### Influence of selenium supplementation on testicular metal homeostasis

The concentration of toxic and essential metals (Al, V, Cr, Mn, Fe, Co, Cu, Zn, As, Mo Cd, Sb, Tl, and Pb) has been determined in the testicles of mice from the different groups to evaluate the metal homeostasis. The results are presented in Table 1. The concentrations of Cd, Sb, Tl and Pb in the testicles were all below the limit of detection (0.02–0.05 ng g^−1^) in all mice groups.

**Table 1:** Concentration of elements (ng g^−1^) expressed as mean ± SD (n = 3) in mice testicles from the different groups.

Element	Control	C-Se	Abx	Abx + Se
Al	1337 ± 380	1239 ± 31	1196 ± 75	829 ± 54
V	9.3 ± 7.3	6.9 ± 0.2	4.5 ± 1.3	6.4 ± 0.2
Cr	65 ± 8	161 ± 4	33 ± 0.2	20 ± 6
Mn	467 ± 16	470 ± 3	448 ± 10	463 ± 1
Fe	31,843 ± 147	25,557 ± 44	28,281 ± 266	32,312 ± 96
Co	17.1 ± 6.7	13.8 ± 1.7	16.3 ± 1.7	21.6 ± 0.8
Cu	1702 ± 17	1429 ± 12	1426 ± 16	1482 ± 30
Zn	3815 ± 31	3662 ± 2	3723 ± 1	4143 ± 42
As	15.4 ± 1.6	40.2 ± 2.7	2.9 ± 1.1	2.4 ± 0.7
Mo	63 ± 4	54 ± 3	130 ± 4	68 ± 1

The statistical analysis showed numerous differences in the concentration of metals between groups. The significant differences for each comparison are summarized in Table S2. Se-supplementation of conventional mice (C-Se vs C) increased the levels of Cr (*p* = 0.004) and As (*p* = 0.008) and decreased the levels of Fe (*p* = 0.000), Cu (*p* = 0.003) and Zn (*p* = 0.020) in the testicles. The apparent paradox of Se increasing the As concentration in the testicles but contributing to As detoxification can be explained by the ability of Se to additionally increase the concentration of betaine in testicles, which sequester As in the non-toxic form of arsenobetaine^55^. The toxicity of Cr is determined by its chemical form, Cr(III) is essential while Cr(VI) is carcinogenic^56^. The effect of Se on metal homeostasis in the testicles was different after microbiota depletion. The concentration of Cr (*p* = 0.023), Cu (*p* = 0.013) and As (*p* = 0.009) were lower in the Abx + Se group when compared to the control group, while the concentration of Zn was higher (*p* = 0.013). However, when Abx + Se is compared with C-Se, the levels of Fe (*p* = 0.000), Co (*p* = 0.028), Zn (*p* = 0.004) and Mo (*p* = 0.024) increased and the levels of Al (*p* = 0.012), Cr (*p* = 0.001) and As (*p* = 0.003) decreased. Finally, Se-supplementation in microbiota depleted mice (Abx + Se vs Abx) reduces the concentrations of Al (*p* = 0.031) and Mo (*p* = 0.003) in the testicles whereas the concentration of Fe (*p* = 0.002) and Zn (*p* = 0.005) were augmented. The cytosol of most eukaryotic cells contains the enzyme superoxide dismutase (SOD), which contains Cu and Zn. After exposure to antibiotics, tissues are subjected to oxidative stress, which likely led to an increase in SOD and therefore, increased Zn levels^57,58^.

Essential metals like Mn, Cu and Zn are crucial for maintaining male reproductive functions, as they are involved in spermatogenesis and sperm motility^59,60^. Moreover, their interaction with toxic elements (As, Cd, Hg, Pb, and others) may change the toxicity of these metals^61^. The synergistic/antagonistic interactions between elements through metal traffic and homeostasis in the different organs and tissues have been reported^62^. The antagonistic role of Se has been proven with toxic elements such as Hg^63^, Cd^64^ and As^65^ and also with organic pollutants^66^.

### Selenoproteins and Gut Microbiota

It is well-known that gut microbiota play important roles in host health, modulating physiological, immunological and metabolic functions, but they also participate in the regulation of hormones related to reproductive functions^16^ through the hypothalamic-pituitary–testicular axis^67^. Recently, the subject of the effects of dietary and supplemented Se on gut microbiota is has been receiving growing attention, but the interplay between testicular selenoproteins and microbiota has not been previously reported. As detailed elsewhere^28^, Se-supplementation shape the gut microbiota composition as well as the effect of the antibiotics treatment (Fig. S3). In brief, Se-supplemented groups showed an increase in members of the *Lachnospiraceae* and *Ruminococcaceae* families as well as *Christensenellaceae* family and *Lactobacillus* genus.

**Figure 4:**
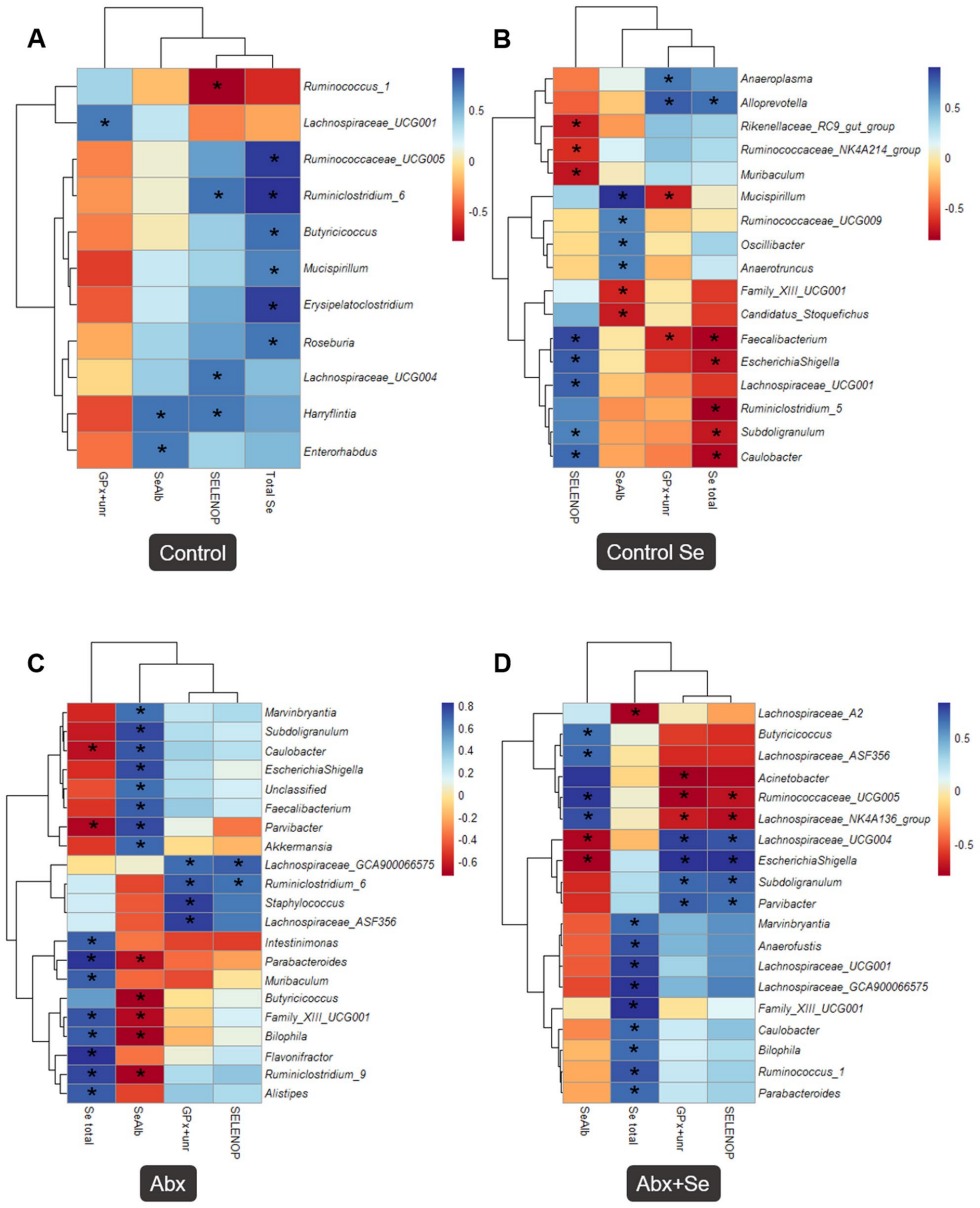
Heatmap showing the correlations between the testicular selenoproteome, total Se content and gut microbiota composition at genus level in the groups: Control (**A**), C-Se (**B**), Abx (**C**), Abx + Se (**D**). The colors range from blue (positive correlation) to red (negative correlation) and (*) indicates a *p-*value ≤ 0.05.

The microbial richness (Chao1 index) and diversity (Shannon index) indeces were associated with selenoproteins in the testicles despite the impact of Abx on the microbial composition. In control group, higher microbial diversity (R = 0.67, *p* ≤ 0.05) and richness (R = 0.71, *p* ≤ 0.05) were associated with higher levels of SeAlb. In the Se-supplemented group, higher microbial diversity was also associated to SeAlb (R = 0.81, *p* ≤ 0.05), but not with microbial richness. Furthermore, the relationship between SeAlb and microbial diversity disappear after the Abx treatment; with one exception: in the Abx + Se group, Chao1 index showed a positive association with SELENOP (R = 0.64, *p* ≤ 0.05). These data suggest a potential effect of the microbiota on the specific selenoproteins in mice testes. To further explore the gut microbiota-testicular selenoproteome interplay, specific associations at the genus level in each group were determined (Fig. 4). The associations at phylum and family levels are detailed in Tables S3–S6.

As shown in Fig. 4, an elevated number of correlations between selenoproteins in the testicles and microbiota composition appeared after Se-supplementation of conventional mice (C-Se) and/or microbiota depleted mice (Abx + Se). It is also noteworthy that the correlations between total Se and SELENOP with microbiota were highly similar in the control mice, and always in the opposite manner than those of GPx + unr. Moreover, this behavior was dependent on the group/treatment. As previously discussed, these findings suggest that the effect of Se-supplementation on the selenoproteome of testicles is influenced by microbiota. In the control group, a higher total Se concentration was positively linked to several groups from the *Ruminococcaceae* and *Lachnospiraceae* families as well as the *Butyricoccus* genus, all known as short chain fatty acids (SCFAs) producers with beneficial impacts on intestinal homeostasis and promoting health benefits. Furthermore, members from the *Lachnospiraceae* and *Ruminococcaceae* families were also found to be associated with testicular functions^68^. Indeed, after the supplementation with Se, some SCFA producers such as *Faecalibacterium* genus as well as *Lachnospiraceae_UCG001* were correlated with SELENOP and *Ruminococceae_UCG009* was correlated with SeAlb. Moreover, in the Abx group these correlations were lost and most of the relations between microbiota and selenoproteome components were found with the total Se and SeAlb (Tables S4–S7). However, in the Abx + Se group, several new associations between all components of selenoproteome were observed, including positive associations between previously mentioned families such as *Lachnospiraceae* groups and *Ruminococcus_1* with Total Se and SeAlb (Fig. 4). It is noteworthy that in the control group these bacteria correlated positively with total Se, while in Se-supplemented mice groups they correlated with specific selenoproteins such as *Mucispirillim*. This observation may indicate that Se-supplementation aids specific functions such as transport (SeAlb and SELENOP) or sperm quality and male fertility (SELENOP)^38,46^.

A higher relative abundance of the *Mucispirillum* genus was associated with higher total Se content in the control group. However, after Se-supplementation, higher relative abundance of this genus was associated with lower GPx + unr and higher SeAlb concentrations in the testicular tissue of conventional mice. A decrease in the level of this genus in mice fed with supranutritional Se has been previously reported^69^. Other authors indicated numerous associations between bacterial taxa and testicular function, but specially showed that *Mucispirillum* were positively correlated with testosterone and sperm activity^68^. *Escherichia/Shigella* has been related with sex hormones in the reproductive endocrine system^70^. This genus correlated positively with SELENOP and negatively with total Se in conventional mice after Se supplementation, while correlating positively with SeAlb in the Abx group. After Se supplementation of this group (Abx + Se), *Escherichia/Shigella* correlated positively with GPx + unr and SELENOP, and negatively with SeAlb.

In summary, Se-supplementation has an impact on the selenoproteome and mineral homeostasis in the testes, and also, on the gut microbiota, suggesting a pivotal key interplay between Se-microbiota and male reproductive health. The metallomic analytical approach, based on the quantification of selenoproteins using an atomic spectrometric detector such as ICP-MS coupled to HPLC, allowed for the first time the absolute quantification of the selenoproteins containing most of the bonded Se in the testicles as well as the novel identification of SeAlb in testicles. Our data indicate that Se-supplementation of conventional mice did not change the total level of Se in the testicles, but significantly changed the selenoproteome profile. Moreover, the opposite situation was observed in microbiota depleted mice suggesting that the effect of Se-supplementation on the selenoproteome of the testicles could be influenced by microbiota. Specific associations between selenoproteins in the testicles and gut microbiota composition and diversity have been observed, some of them related to sperm activity and sex hormones, demonstrating the interplay of Se supplementation with microbiota and the impact on reproductive health. More studies are needed to ascertain the mechanisms behind the Se-microbiota-reproductive health.

## Materials and methods

### Animal experimental design

After a three day acclimation period, forty mice (male *Mus musculus* BALB/c, 8 weeks old) were randomly divided into two groups, one receiving water and the other, receiving water with a mixture of antibiotics (ampicillin 1%, metronidazole 1%, neomycin 1%, vancomycin 0.5%) and an antifungal (amphotericin B, 10 mg/L) for one week. After this pretreatment time, half of the mice in each group (n = 10) were fed for an additional two weeks (treatment period) with the same regular diet used in the previous days, and the other half, were fed with a Se-enriched diet (0.65 mg/kg of sodium selenite). The four groups, C (control), C-Se (Se-enriched diet during treatment); Abx (antibiotics in the water during pretreatment) and Abx + Se (Antibiotics in the water during pretreatment and Se-enriched diet during treatment) were caged in pairs, with free access to water and food, which were changed every other day. Figure S1 summarizes the design of the experiment. At the end of the experiment, mice were anesthetized (isoflurane) and sacrificed by cervical dislocation, and organs were immediately removed, cleaned in NaCl (0.9% w/w) solution, cryo-homogenized in liquid nitrogen and stored at -80ºC until analysis.

### Ethics statement

The experimental procedures were carried out at the Animal Experimentation Service of the University of Cordoba (SAEX-UCO), after approval by the bioethics committee of the university and the regional government (Code Num. 02-01-2019-001), in accordance with current European Union regulations. Furthermore, the study was carried out in compliance with the ARRIVE Guideline.

### Histopathological evaluation

Testicle tissues were fixed in 10% neutral buffered formalin for 24 h and then embedded in paraffin wax. 4 µm-thick paraffin serial sections were obtained using a rotary microtome (SAKURA Tissue Tek Accu Cut SRM 119 200) and stained with hematoxylin–eosin according to routine protocols. Photomicrographs were obtained with a Nikon Eclipse E400 photomicroscope at 400 magnifications.

### Speciation of selenoproteins in mice testicles

To isolate selenoproteins, testicles were cryo-homogenized with a mortar and pestle in the presence of liquid nitrogen. Selenoproteins were extracted using the CelLytic™ MT extraction reagent (Sigma-Aldrich, Steinheim, Germany) (3 Ml g^−1^) into a glass/teflon homogenizer at 4 °C. Protease inhibitor cocktail (Sigma-Aldrich, Steinheim, Germany) was added to CelLytic MT reagent to avoid protein degradation. Then, the mixture was centrifuged at 15,500*g* for 20 min at 4 °C. The supernatant was collected and filtered through low protein absorption Iso-Disc poly(vinylidene difluoride) filters (PVDF, 25 mm diameter, 0.45 µm pore size). A preconcentration step is necessary due to the low concentration of selenospecies. To this end, the extracts were completely evaporated under a nitrogen stream and re-dissolved in 0.1 mL of MilliQ water prior to the analysis.

Selenoproteins were separated from the obtained extracts using a previously described method^71^. Briefly, the chromatographic separation of GPx, SELENOP and SeAlb were performed with an ultra-high performance liquid chromatograph (model 1260 Infinity Quaternary LC, Agilent Technologies) using two size exclusion columns (5 ml HiTrap ®Desalting Columns, GE Healthcare, Uppsala, Sweden) and two different affinity chromatography columns (AFC, GE Healthcare, Uppsala, Sweden) with stationary phases of heparine-sepharose column (HEP-HP) and blue-sepharose column (BLUE-HP). The HEP-HP column retains only SELENOP, while the BLUE-HP column retains SELENOP and SeAlb. The column switching method allows the simultaneous separation of selenoproteins and selenometabolites: GPx and selenometabolites elute when the column switching system is in position 1 (0–20 min), while SELENOP and SeAlb are retained in the AFC columns. The valve switches to position 2 (20–24 min) to elute the SELENOP, and then returns to position 1 for the elution of SeAlb. Polyether ether ketone (PEEK) tubing (30 cm × 0.6 mm i.d.) and a T-connector were used to connect the eluent of the chromatograph to the Micromist nebulizer (Glass Expansion, Switzerland) of the triple quadrupole inductively coupled plasma mass spectrometer (ICP-QqQ-MS, model Agilent 8800 Triple Quad, Agilent Technologies, Tokyo, Japan) (2D-SEC-SEC-AFxAF-ICP-MS)^74^. Se (Cambridge Isotope Laboratories, Andover, MA, USA) was also introduced into the system via a T-connector for isotope dilution analysis. The absolute quantification of selenoproteins by 2D-SEC-AF-SUID-ICP-MS was carried out using the operational conditions as summarized in Table S7.

### Total elements determination in mice testicles

For total elemental analysis, testicular tissue samples from mice in each group were pooled, and approximately, 0.1000 g of sample were digested in a microwave reaction system MARS 6 (CEM Corporation, Matthews, NC, USA) with a mixture of nitric acid and hydrogen peroxide (4:1, v/v). The mineralization was carried out from room temperature to 160 °C over 15 min, then maintaining at 400 W for 40 min. Then, the samples were diluted fivefold in 5% HNO_3_ containing 100 µg L^−1^ of rhodium, and filtered using 0.45 µm PTFE syringe filters prior to the analysis by ICP-QqQ-MS. The operational conditions for ICP-QqQ-MS are listed in Table S7. The validation of the methodology was carried out using a fish protein certified reference material for trace element DORM-4 (National Research Council of Canada) (Table S8).

### Gut microbiota analysis

Fecal samples were collected from the colon and immediately frozen in liquid nitrogen. DNA from fecal samples (approx. 100 mg) was obtained with the Master-Pure DNA extraction kit (Epicentre, Madison, WI, United States) following the manufacturer’s instructions. Specific modifications were included as described elsewhere^72^. DNA concentration was measured using a Qubit® 2.0 Fluorometer (Life Technology, Carlsbad, CA, United States). A specific 16S rRNA amplicon (V3-V4 variable region of the 16S rRNA gene) was amplified and sequenced following Illumina protocols. Briefly, a multiplexing step was conducted using the NextEra Index Kit (Illumina, San Diego, CA, United States) and amplicons were checked with a Bioanalyzer DNA 1000 chip (Agilent Technologies, Santa Clara, CA, United States). Libraries were sequenced (2 × 300 bp paired-end run, MiSeq Reagent kit v3) on a MiSeq-Illumina platform (FISABIO sequencing service, Valencia, Spain). Controls (DNA extraction procedure and libraries amplification) were included. A DADA2 pipeline was used to achieve quality filtering, sequence joining and chimera removal^73^. Taxonomy assignment was performed using Silva v132 database^74,75^. Sample with less than 1000 reads as well as specific taxa present at levels less than 0.01% and those present less than 3 times in at least 20% of the samples were filtered and removed from the analysis. Furthermore, sequences classified as Chloroplast and Cyanobacteria were filtered from the final dataset as they are associated with potential contaminants.

### Statistical analysis

Statistical analysis was performed using Minitab16 Statistical Software (State College, PA, United States) and STATISTICA 8 Software. Firstly, Anderson–Darling normality test was used to determine whether or not data are normally distributed. Differences between groups were tested using the Kruskal–Wallis test (non-parametric statistics) and one-way ANOVA (parametric statistics). The Spearman correlation test was performed for correlation analysis between gut microbiota abundance and selenoproteins concentrations. The level of *p* < 0.05 was considered statistically significant. Heatmaps were generated in R Project software (version 4.0.2) (R Core Team (2020). R: A language and environment for statistical computing. R Foundation for Statistical Computing, Vienna, Austria). For the microbiota analyses, total sum normalization (TSS) for the statistical analysis, multivariate test and data mining were performed with Calypso web platform v. 8.56^76^. Alpha- diversity metrics (Chao1 and Shannon indeces) and beta diversity analysis (based on Bray Curtis distance) were obtained. Briefly, Permutational multivariate analysis of variance (ADONIS) and Redundancy Discriminant Analysis (RDA) were obtained. Relative abundance (%) differences between groups at different taxonomical levels were tested using the Krustal-Wallis test with False discovery test rate (FDR) for multiple test correction. Alpha diversity indexes were obtained after a rarefaction to 93,525 sequences (minimum number of reads per sample). The level of statistical significance for all tests was fixed to p < 0.05.

## Supplementary Information

Supplementary Information.
