# Supplementary material for: Selenium supplementation influences mice testicular selenoproteins driven by gut microbiota
Source: Sci Rep. 2022 Mar 10;12:4218. doi: 10.1038/s41598-022-08121-3 (PMC8913620; doi:10.1038/s41598-022-08121-3)
Supplement: Supplementary file 1 — Supplementary Information. [file 41598_2022_8121_MOESM1_ESM.docx]

**SUPPORTING INFORMATION**

**Selenium supplementation influences mice testicular selenoproteins driven by gut microbiota**

**Sara Ramírez-Acosta^1^, Marta Selma-Royo^2^, María C. Collado**^∑^**^2^, Francisco Navarro Roldán**^∑^**^3^, Nieves Abril**^∑^**^4^, Tamara García-Barrera**^∑^**^1*^**

^1^Research Center of Natural Resources, Health and the Environment (RENSMA). Department of Chemistry, Faculty of Experimental Sciences, Campus El Carmen, University of Huelva, Fuerzas Armadas Ave., 21007, Huelva, Spain

^2^Institute of Agrochemistry and Food Technology-National Research Council (IATA-CSIC), Department of Biotechnology, Agustin Escardino 7. 46980 Paterna, Valencia, Spain

^3^Department of Integrated Sciences, Cell Biology, Faculty of Experimental Sciences, University of Huelva, Spain;

^4^Department of Biochemistry and Molecular Biology, University of Córdoba, Campus de Rabanales, Edificio Severo Ochoa, E-14071, Córdoba, Spain.

**^*^**tamara@dqcm.uhu.es

^∑^Senior authors


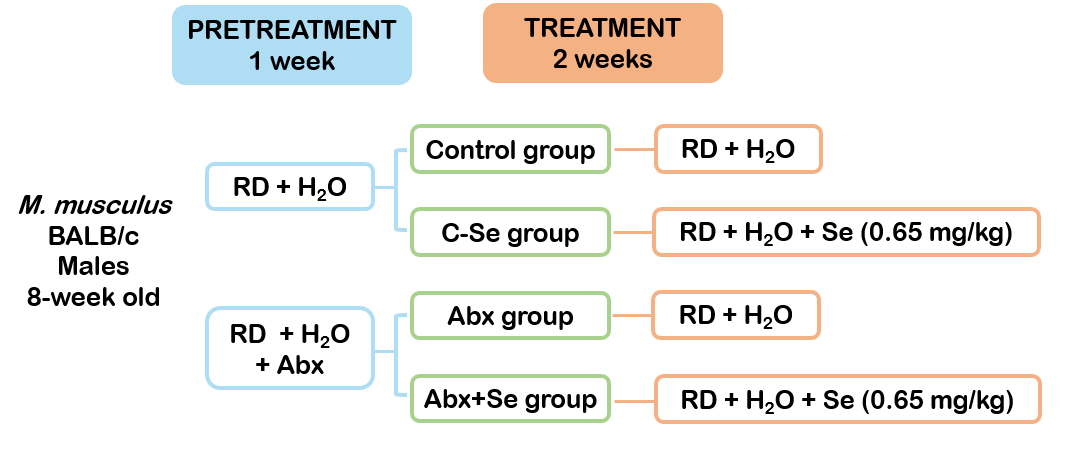


**Figure S1**. Experimental design. Abbreviations: RD: regular diet, Abx: antibiotics (ampicillin 1%, metronidazole 1%, neomycin 1%, vancomycin 0.5%; amphotericin B 10 mg/L).


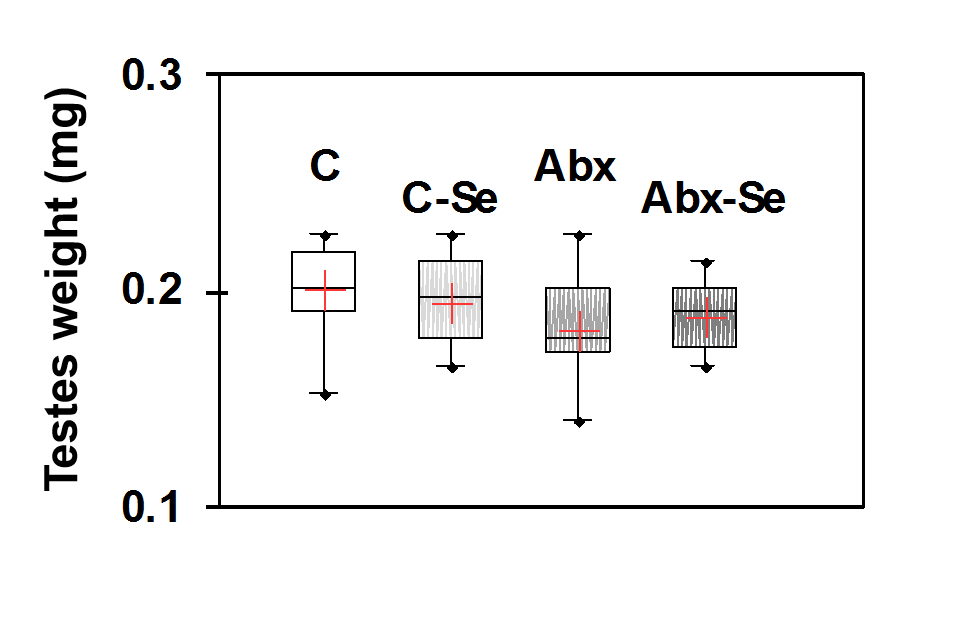


**Figure S2**. Box-plots showing the differences in testes weight in the four experimental groups at the end of the treatment. The red crosses correspond to the means. The line in the middle of each box represents the median for each group examined, and the edges of the box represent the first (lower limit) and the third (upper limit) quartiles. Points above or below the upper and lower limits of the whiskers can be considered outliers.


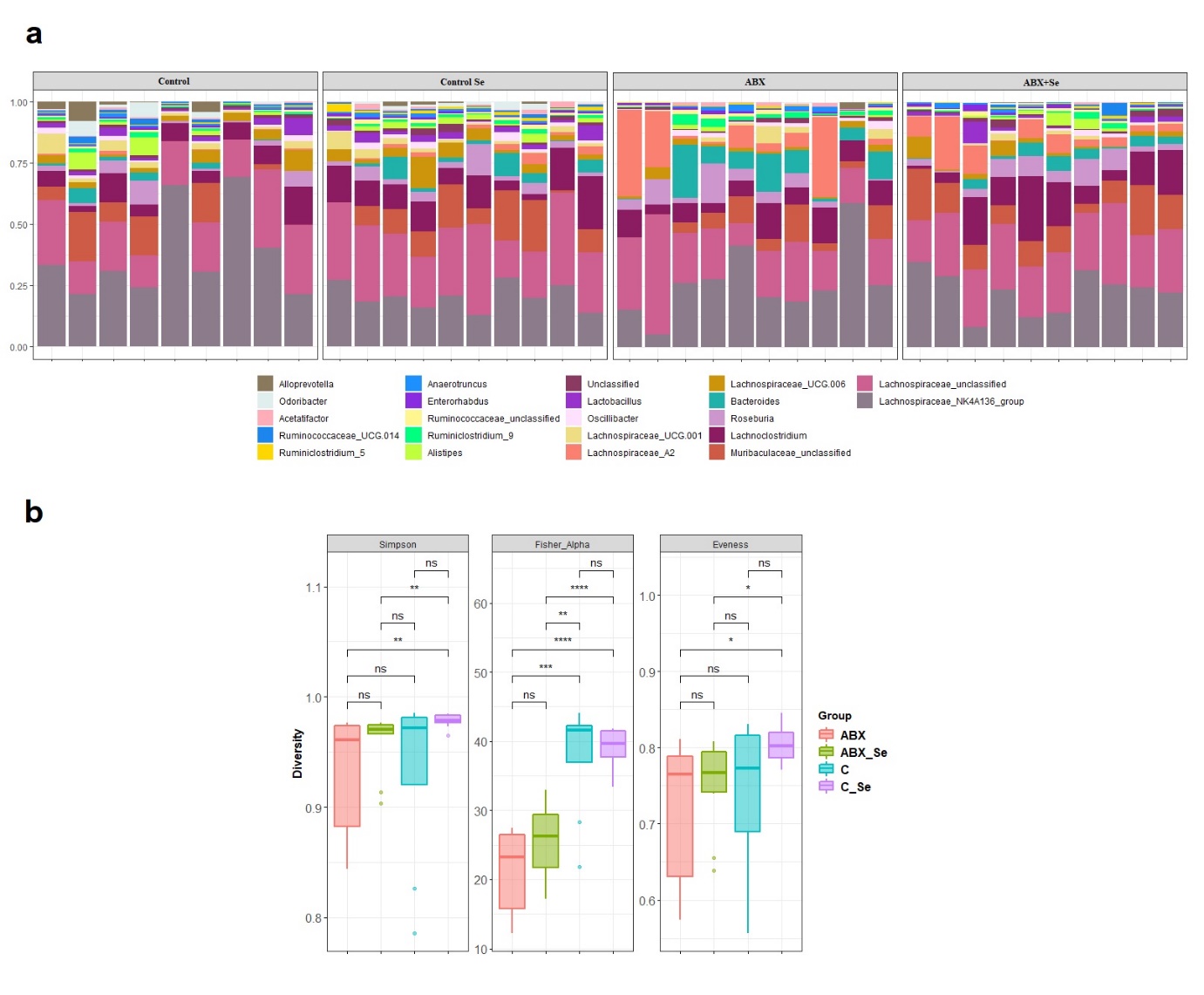


**Figure S3. Description of the gut microbiota composition in the experimental groups.** **A)** Bar plots showing the composition of gut microbiota at genus level facet by experimental group. Only those genera with a relative abundance >0.1% are shown in the figure. Unclassified groups from Lachnospiraceae, Ruminococcaceae and Muribaculaceae families were also plotted due to its importance for the general composition. **B)** Alpha diversity of the gut microbiota of the experimental groups measured as Simpson, Fisher-alpha and Eveness indexes. ABX: Antibiotic treated group, ABX_Se: Se supplementation after antibiotic treatment, C: Control group, C_Se: Se supplemented group. *p<0.05, **p<0.01, ***p<0.001, ****p<0.0001.

|  | **GPx+unr** | | **SELENOP** | | **SeAlb** | | **Total Se** | |
| --- | --- | --- | --- | --- | --- | --- | --- | --- |
| **Groups** | Fold change | *p*-value | Fold change | *p*-value | Fold change | *p*-value | Fold change | *p*-value |
| CSe *vs* C | 0.43 | 0.000 | 1.61 | 0.000 | 1.30 | 0.013 | N.S | |
| Abx+Se *vs* C | N.S | | N.S | | 0.65 | 0.017 | 1.24 | 0.001 |
| Abx+Se *vs* CSe | 2.22 | 0.000 | 0.54 | 0.000 | 0.50 | 0.000 | 1.19 | 0.005 |
| Abx+Se *vs* Abx | N.S | | N.S | | N.S | | 1.15 | 0.010 |

**Table S1.** Significant changes in the concentration of total selenium and selenoproteins in testicles. N.S., non-significant.

|  | **CSe *vs* C** | | **Abx+Se *vs* C** | | **Abx+Se *vs* CSe** | | **Abx+Se *vs* Abx** | |
| --- | --- | --- | --- | --- | --- | --- | --- | --- |
| **Element** | Fold change | *p*-value | Fold change | *p*-value | Fold change | *p*-value | Fold change | *p*-value |
| Al | N.S. | N.S. | N.S. | N.S. | 0.67 | 0.012 | 0.69 | 0.031 |
| V | N.S. | N.S. | N.S. | N.S. | N.S. | N.S. | N.S. | N.S. |
| Cr | 2.48 | 0.004 | 0.31 | 0.023 | 0.12 | 0.001 | N.S. | N.S. |
| Mn | N.S. | N.S. | N.S. | N.S. | N.S. | N.S. | N.S. | N.S. |
| Fe | 0.80 | 0.000 | N.S. | N.S. | 1.26 | 0.000 | 1.14 | 0.002 |
| Co | N.S. | N.S. | N.S. | N.S. | 1.56 | 0.028 | N.S. | N.S. |
| Cu | 0.84 | 0.003 | 0.87 | 0.013 | N.S. | N.S. | N.S. | N.S. |
| Zn | 0.96 | 0.020 | 1.09 | 0.013 | 1.13 | 0.004 | 1.11 | 0.005 |
| As | 2.61 | 0.008 | 0.16 | 0.009 | 0.06 | 0.003 | N.S. | N.S. |
| Mo | N.S. | N.S. | N.S. | N.S. | 1.28 | 0.024 | 0.53 | 0.003 |

**Table S2.** Elements statistically different for each comparison. N.S.: non-significant.

| **CONTROL** | | | |
| --- | --- | --- | --- |
| **Bacteria** | **Se** | **Correlation** | **p-value** |
| Deferribacteres | Total Se | 0.67 | 0.048 |
| Actinobacteria | SeAlb | 0.764 | 0.017 |
| *Eggerthellaceae* | SeAlb | 0.7625 | 0.017 |
| *Deferribacteraceae* | Total Se | 0.67 | 0.048 |
| *Christensenellaceae* | SELENOP | 0.6986 | 0.036 |
| *Ruminococcus_1* | SELENOP | -0.8016 | 0.009 |
| *Ruminococcaceae_UCG005* | Total Se | 0.882 | 0.002 |
| *Ruminiclostridium_6* | SELENOP | 0.7161 | 0.03 |
|  | Total Se | 0.912 | 0.001 |
| *Roseburia* | Total Se | 0.71 | 0.032 |
| *Mucispirillum* | Total Se | 0.67 | 0.048 |
| *Lachnospiraceae_UCG004* | SELENOP | 0.7013 | 0.035 |
| *Lachnospiraceae_UCG001* | GPx+unr | 0.6979 | 0.037 |
| *Harryflintia* | SeAlb | 0.715 | 0.03 |
|  | SELENOP | 0.7015 | 0.035 |
| *Erysipelatoclostridium* | Total Se | 0.8711 | 0.002 |
| *Enterorhabdus* | SeAlb | 0.6964 | 0.037 |
| *Butyricicoccus* | Total Se | 0.7298 | 0.026 |

**Table S3**. Correlation between the gut microbiota and testicular selenoproteome and total Se content in control group.

| **CONTROL Se** | | | |
| --- | --- | --- | --- |
| **Bacteria** | **Se** | **Correlation** | **p-value** |
| Tenericutes | GPx+unr | 0.6758 | 0.032 |
| Deferribacteres | GPx+unr | -0.682 | 0.03 |
|  | SeAlb | 0.8901 | 0.01 |
| *Unclassified* | SELENOP | 0.7458 | 0.013 |
| *Streptococcaceae* | SELENOP | 0.7268 | 0.017 |
|  | Total Se | -0.736 | 0.015 |
| *Prevotellaceae* | GPx+unr | 0.6775 | 0.031 |
|  | Total Se | 0.6408 | 0.046 |
| *Family_XIII* | SeAlb | -0.7484 | 0.013 |
| *Enterobacteriaceae* | SELENOP | 0.7457 | 0.013 |
|  | Total Se | -0.7255 | 0.018 |
| *Deferribacteraceae* | GPx+unr | -0.6822 | 0.03 |
|  | SeAlb | 0.8902 | 0.001 |
| *Clostridiaceae_1* | GPx+unr | -0.8345 | 0.003 |
|  | SELENOP | 0.7002 | 0.024 |
| *Christensenellaceae* | SELENOP | -0.933 | 0.000 |
| *Caulobacteraceae* | Total Se | -0.6999 | 0.024 |
| *Anaeroplasmataceae* | GPx+unr | 0.6761 | 0.032 |
| *Subdoligranulum* | SELENOP | 0.6453 | 0.044 |
|  | Total Se | -0.717 | 0.02 |
| *Ruminococcaceae_UCG009* | SeAlb | 0.6345 | 0.049 |
| *Ruminococcaceae_NK4A214_group* | SELENOP | -0.6335 | 0.045 |
| *Ruminiclostridium_5* | Total Se | -0.8294 | 0.003 |
| *Rikenellaceae_RC9_gut_group* | SELENOP | -0.6884 | 0.028 |
| *Oscillibacter* | SeAlb | 0.6484 | 0.043 |
| *Muribaculum* | SELENOP | -0.7285 | 0.017 |
| *Mucispirillum* | GPx+unr | -0.6825 | 0.03 |
|  | SeAlb | 0.8902 | 0.001 |

**Table S4**. Correlation between the gut microbiota and testicular selenoproteome and total Se content in CSe group.

| **CONTROL Se** | | | |
| --- | --- | --- | --- |
| **Bacteria** | **Se** | **Correlation** | **p-value** |
| *Family_XIII_UCG001* | SeAlb | -0.6649 | 0.036 |
| *Faecalibacterium* | GPx+unr | -0.6697 | 0.034 |
|  | SELENOP | 0.8219 | 0.004 |
|  | Total Se | -0.7994 | 0.006 |
| *EscherichiaShigella* | SELENOP | 0.7642 | 0.01 |
|  | Total Se | -0.7314 | 0.016 |
| *Caulobacter* | SELENOP | 0.7196 | 0.019 |
|  | Total Se | -0.7795 | 0.008 |
| *Candidatus_Stoquefichus* | SeAlb | -0.6993 | 0.021 |
| *Anaerotruncus* | SeAlb | 0.6475 | 0.043 |
| *Anaeroplasma* | GPx+unr | 0.6761 | 0.032 |
| *Alloprevotella* | GPx+unr | 0.7556 | 0.011 |
|  | Total Se | 0.708 | 0.022 |

**Table S4** (continued). Correlation between the gut microbiota and testicular selenoproteome and total Se content in CSe group.

| **ABX** | | | |
| --- | --- | --- | --- |
| **Bacteria** | **Se** | **Correlation** | **p-value** |
| Verrucomicrobia | SeAlb | 0.6689 | 0.034 |
| Firmicutes | SeAlb | 0.7239 | 0.018 |
| Bacteroidetes | SeAlb | -0.7207 | 0.019 |
| *Unclassified* | SeAlb | 0.7091 | 0.022 |
| *Tannerellaceae* | SeAlb | -0.6536 | 0.04 |
|  | Total Se | 0.8127 | 0.004 |
| *Staphylococcaceae* | GPx+unr | 0.7821 | 0.008 |
| *Ruminococcaceae* | SeAlb | -0.7545 | 0.012 |
|  | Total Se | 0.7167 | 0.02 |
| *Lachnospiraceae* | SeAlb | 0.7274 | 0.018 |
|  | Total Se | -0.6394 | 0.047 |
| *Enterobacteriaceae* | SeAlb | 0.7175 | 0.019 |
| *Desulfovibrionaceae* | SeAlb | -0.7357 | 0.015 |
|  | Total Se | 0.7123 | 0.021 |
| *Clostridiaceae_1* | SeAlb | 0.6947 | 0.026 |
| *Caulobacteraceae* | SeAlb | 0.7164 | 0.02 |
|  | Total Se | -0.6591 | 0.038 |
| *Burkholderiaceae* | SeAlb | 0.7747 | 0.009 |
|  | Total Se | -0.6467 | 0.043 |
| *Akkermansiaceae* | SeAlb | 0.6689 | 0.034 |
| *Unclassified* | SeAlb | 0.6504 | 0.042 |
| *Subdoligranulum* | SeAlb | 0.7536 | 0.012 |
| *Staphylococcus* | GPx+unr | 0.7821 | 0.008 |
| *Ruminiclostridium_9* | SeAlb | -0.7192 | 0.019 |
|  | Total Se | 0.7641 | 0.01 |
| *Ruminiclostridium_6* | GPx+unr | 0.7081 | 0.022 |
|  | SELENOP | 0.6474 | 0.043 |
| *Parvibacter* | SeAlb | 0.7476 | 0.013 |
|  | Total Se | -0.6931 | 0.026 |
| *Parabacteroides* | SeAlb | -0.6536 | 0.04 |
|  | Total Se | 0.8127 | 0.004 |

**Table S5**. Correlation between the gut microbiota and testicular selenoproteome and total Se content in Abx group.

| **ABX** | | | |
| --- | --- | --- | --- |
| **Bacteria** | **Se** | **Correlation** | **p-value** |
| *Muribaculum* | Total Se | 0.6968 | 0.025 |
| *Marvinbryantia* | SeAlb | 0.6538 | 0.04 |
| *Intestinimonas* | Total Se | 0.6882 | 0.028 |
| *Lachnospiraceae_GCA900066575* | GPx+unr | 0.6603 | 0.038 |
|  | SELENOP | 0.6967 | 0.025 |
| *Flavonifractor* | Total Se | 0.8197 | 0.004 |
| *Family_XIII_UCG001* | SeAlb | -0.6821 | 0.03 |
|  | Total Se | 0.7272 | 0.017 |
| *Faecalibacterium* | SeAlb | 0.7024 | 0.024 |
| *EscherichiaShigella* | SeAlb | 0.7466 | 0.013 |
| *Caulobacter* | SeAlb | 0.7164 | 0.02 |
|  | Total Se | -0.6591 | 0.038 |
| *Butyricicoccus* | SeAlb | -0.7341 | 0.016 |
| *Bilophila* | SeAlb | -0.7332 | 0.016 |
|  | Total Se | 0.71 | 0.021 |
| *Lachnospiraceae_ASF356* | GPx+unr | 0.7821 | 0.008 |
| *Alistipes* | Total Se | 0.708 | 0.022 |
| *Akkermansia* | SeAlb | 0.6689 | 0.034 |

**Table S5** (continued). Correlation between the gut microbiota and testicular selenoproteome and total Se content in Abx group.

| **ABX+Se** | | | |
| --- | --- | --- | --- |
| **Bacteria** | **Se** | **Correlation** | **p-value** |
| *Proteobacteria* | SELENOP | 0.6479 | 0.043 |
| *Unclassified* | GPx+unr | 0.7233 | 0.018 |
|  | SELENOP | 0.8523 | 0.002 |
|  | SeAlb | -0.8345 | 0.003 |
| *Tannerellaceae* | Total Se | 0.6573 | 0.039 |
| *Peptococcaceae* | Total Se | 0.6515 | 0.041 |
| *Moraxellaceae* | GPx+unr | -0.8017 | 0.005 |
|  | SELENOP | -0.7519 | 0.012 |
|  | SeAlb | 0.805 | 0.005 |
| *Family_XIII* | Total Se | 0.7569 | 0.011 |
| *Eubacteriaceae* | Total Se | 0.7286 | 0.017 |
| *Enterobacteriaceae* | GPx+unr | 0.7653 | 0.01 |
|  | SELENOP | 0.7875 | 0.007 |
|  | SeAlb | -0.7414 | 0.014 |
| *Desulfovibrionaceae* | Total Se | 0.6536 | 0.04 |
| *Clostridiaceae_1* | GPx+unr | 0.7045 | 0.023 |
|  | SELENOP | 0.6639 | 0.036 |
| *Subdoligranulum* | GPx+unr | 0.6652 | 0.036 |
|  | SELENOP | 0.6909 | 0.027 |
| *Ruminococcus_1* | Total Se | 0.7149 | 0.02 |
| *Ruminococcaceae_UCG005* | GPx+unr | -0.7799 | 0.008 |
|  | SELENOP | -0.713 | 0.021 |
|  | SeAlb | 0.7964 | 0.006 |
| *Parvibacter* | GPx+unr | 0.6844 | 0.029 |
|  | SELENOP | 0.6426 | 0.045 |
| *Parabacteroides* | Total Se | 0.6573 | 0.039 |
| *Marvinbryantia* | Total Se | 0.6575 | 0.039 |
| *Lachnospiraceae_UCG004* | GPx+unr | 0.7732 | 0.009 |
|  | SELENOP | 0.7214 | 0.019 |
|  | SeAlb | -0.7612 | 0.011 |
| *Lachnospiraceae_UCG001* | Total Se | 0.7702 | 0.009 |

**Table S6**. Correlation between the gut microbiota and testicular selenoproteome and total Se content in Abx + Se group.

| **ABX+Se** | | | |
| --- | --- | --- | --- |
| **Bacteria** | **Se** | **Correlation** | **p-value** |
| *Lachnospiraceae_NK4A136_group* | GPx+unr | 0.7732 | 0.009 |
|  | SELENOP | 0.7214 | 0.019 |
|  | SeAlb | -0.7612 | 0.011 |
| *Lachnospiraceae_GCA900066575* | Total Se | 0.8111 | 0.004 |
| *Family_XIII_UCG001* | Total Se | 0.8185 | 0.004 |
| *EscherichiaShigella* | GPx+unr | 0.8137 | 0.004 |
|  | SELENOP | 0.8185 | 0.004 |
|  | SeAlb | -0.7882 | 0.007 |
| *Caulobacter* | Total Se | 0.6541 | 0.04 |
| *Butyricicoccus* | SeAlb | 0.6467 | 0.043 |
| *Bilophila* | Total Se | 0.6536 | 0.04 |
| *Lachnospiraceae_ASF356* | SeAlb | 0.6616 | 0.037 |
| *Anaerofustis* | Total Se | 0.7286 | 0.017 |
| *Acinetobacter* | GPx+unr | -0.8017 | 0.005 |
|  | SELENOP | -0.7519 | 0.012 |
|  | SeAlb | 0.805 | 0.005 |
| *Lachnospiraceae_A2* | Total Se | -0.7834 | 0.007 |

**Table S6** (continued). Correlation between the gut microbiota and testicular selenoproteome and total Se content in Abx + Se group.

| **ICP-MS operational conditions** | |
| --- | --- |
| Forward power | 1550 W |
| Nebulizer | MicroMist |
| Plasma gas flow rate | 15 L·min^-1^ |
| Carrier gas flow rate | 1.08 L·min^-1^ |
| Reaction gases | 40% O_2_, H_2_ |
| Sampling and skimmer cones | Ni |
| Sampling depth | 10 mm |
| Isotopes monitored | ^27^Al, ^51^V, ^52^Cr, ^55^Mn, ^56^Fe, ^59^Co,^60^Ni, ^63^Cu, ^66^Zn, ^75^As, ^74^Se, ^76^Se, ^77^Se, ^78^Se, ^80^Se ^95^Mo, ^103^Rh, ^111^Cd, ^121^Sb, ^205^Tl, ^208^Pb |
|  |  |
|  |  |
|  |  |
|  |  |
| Integration time | 0.3 s |
| **Chromatographic conditions** | |
| Flow rate | 1.3 mL·min^-1^ |
| Mobile phase A | 0.05 M ammonium acetate |
| Mobile phase B | 1.5 M ammonium acetate |
| Gradient | 0-12 min 0% B |
|  | 12-25 min 100% B |
|  | 25-40 min 0%B |
| Valve position | position 1 (0-20 min) |
|  | position 2 (20-24 min) |
|  | position 1 (24-40 min) |

**Table S7.** Operational conditions for speciation of selenoproteins and total metal content.

|  | **DORM-4 fish protein certified reference material for trace metals (mean±SD)** | |
| --- | --- | --- |
| **Element** | *Obtained value (mg/kg)* | *Certified value (mg/kg)* |
| Al | 1266.33 ± 27.93 | 1280 ± 340 (*) |
| V | 1.47 ± 0.02 | 1.57 ± 0.14 |
| Cr | 1.88 ± 0.004 | 1.87 ± 0.18 |
| Mn | 2.82 ± 0.08 | 3.17 ± 0.26 |
| Fe | 340.87 ± 2.99 | 343 ± 20 |
| Co | 0.27 ± 0.03 | 0.25 (**) |
| Cu | 15.86 ± 0.41 | 15.7 ± 0.46 |
| Zn | 48.29 ± 0.49 | 51.6 ± 2.8 |
| As | 6.95 ± 0.07 | 6.87 ± 0.44 |
| Se | 3.71 ± 0.02 | 3.45 ± 0.4 |
| Mo | 0.32 ± 0.02 | 0.25 (**) |
| Cd | 0.31 ± 0.02 | 0.299 ± 0.018 |
| Tl | 0.0001 ± 0.00004 | - |
| Sb | 0.012 ± 0.001 | - |
| Pb | 0.34 ± 0.001 | 0.404 ± 0.062 |

**Table S8.** Total metal content of CRM DORM-4 and its established certified, reference (*), and information values (**).
